# Overexpression of the Maize *ZmNLP6* and *ZmNLP8* Can Complement the *Arabidopsis* Nitrate Regulatory Mutant *nlp7* by Restoring Nitrate Signaling and Assimilation

**DOI:** 10.3389/fpls.2017.01703

**Published:** 2017-10-05

**Authors:** Huairong Cao, Shengdong Qi, Mengwei Sun, Zehui Li, Yi Yang, Nigel M. Crawford, Yong Wang

**Affiliations:** ^1^State Key Laboratory of Crop Biology, College of Life Sciences, Shandong Agricultural University, Tai’an, China; ^2^Section of Cell and Developmental Biology, Division of Biological Sciences, University of California, San Diego, La Jolla, CA, United States

**Keywords:** Maize, nitrate assimilation, nitrate regulatory gene, nitrate signaling, *ZmNLP6* and *ZmNLP8*

## Abstract

Nitrate is a key nutrient that affects maize growth and yield, and much has yet to be learned about nitrate regulatory genes and mechanisms in maize. Here, we identified nine *ZmNLP* genes in maize and analyzed the functions of two *ZmNLP* members in nitrate signaling. qPCR results revealed a broad pattern of expression for *ZmNLP* genes in different stages and organs with the highest levels of transcript expression of *ZmNLP6* and *ZmNLP8*. When *ZmNLP6* and *ZmNLP8* were overexpressed in the *Arabidopsis* nitrate regulatory gene mutant *nlp7-4*, nitrate assimilation and induction of nitrate-responsive genes in the transgenic plants were recovered to WT levels, indicating that *ZmNLP6* and *ZmNLP8* can replace the essential roles of the master nitrate regulatory gene *AtNLP7* in nitrate signaling and metabolism. ZmNLP6 and ZmNLP8 are localized in the nucleus and can bind candidate nitrate-responsive *cis*-elements *in vitro*. The biomass and yield of transgenic *Arabidopsis* lines overexpressing *ZmNLP6* and *ZmNLP8* showed significant increase compared with WT and *nlp7-4* mutant line in low nitrate conditions. Thus, *ZmNLP6* and *ZmNLP8* regulate nitrate signaling in transgenic *Arabidopsis* plants and may be potential candidates for improving nitrogen use efficiency of maize.

## Introduction

Nitrogen is one of the most critical macronutrients for plant growth, development, and production (Crawford and Forde, 2002; Kusano et al., 2011). Most plants that grow in aerobic soils, such as maize and wheat absorb nitrogen mainly in the form of nitrate. In agriculture, nitrogen fertilizer has been widely used to maintain the high yield of crops. Because of low NUE, nitrate cannot be completely absorbed by plants and therefore a large part is lost to the environment, resulting in serious environmental pollution. Improving NUE is key to solving these problems. However, the current progress in NUE is limited by the fact that genes and mechanisms involved in nitrate regulation in plants are still poorly understood. Thus, identification and characterization of nitrate regulatory genes and underlining mechanisms is of great importance, especially in crop plants, for developing sustainable agriculture.

The uptake and transport of nitrate in plants have been found to be achieved by *NRT1, NRT2, CLC*, and *SLAH* gene families (Krapp et al., 2014). After importation into the cells, nitrate will be reduced then assimilated into amino acids by the action of NR, NiR and GS/glutamate synthase (GOGAT). Nitrate serves not only as a nutrient, but also as a signal. Previous reports on nitrate regulatory genes mainly focused on components involved in regulating root architecture and the PNR in *Arabidopsis* (Alvarez et al., 2012; Forde, 2014; Medici and Krouk, 2014). *AtANR1*, an *Arabidopsis* MADS-box gene, was the first identified nitrate regulator to function in lateral root branching in response to nitrate (Zhang and Forde, 1998). Later, the nitrate transporter AtNRT1.1 was found to work upstream of AtANR1 in regulating lateral root development on nitrate-rich patches (Remans et al., 2006). A unique nitrogen-responsive module AtmiR393/AFB3 controls the growth of both primary and lateral root in response to external and internal nitrogen availability in *Arabidopsis* (Vidal et al., 2010). Recently, AtTCP20 was characterized to redirect root growth to nitrate-rich regions by a systemic signaling pathway and to support root meristem growth under nitrate starvation with the interaction of AtNLP7 (Guan et al., 2014, 2017). In addition, CLE, a peptide generated in the root cells, was identified to interact with AtCLV1 to control the development of lateral root in nitrogen-deficient environment (Araya et al., 2014). Another peptide CEP induced by nitrogen starvation in roots is translocated to shoots to bind CEP receptor to regulate the lateral root foraging (Tabata et al., 2014). AtABI2, a member of protein phosphatase 2C family, dephosphorylates AtCIPK23 and AtCBL1 to modulate nitrate uptake and lateral root development in a AtNRT1.1-dependent manner in *Arabidopsis* (Liu et al., 2015).

During the last several years, a few of the regulatory genes involved in PNR have also been identified in *Arabidopsis*. AtNRT1.1 (NPF6.3), which was previously identified as a dual-affinity nitrate transporter, has been found to be a nitrate sensor (transceptor) to regulate nitrate signaling (Ho et al., 2009; Wang et al., 2009; Gojon et al., 2011). The AtNRT1.1 can be phosphorylated by AtCIPK23 to respond to different nitrate concentrations (Ho et al., 2009). Furthermore, *AtNRG2* has been isolated by a forward genetics approach and functions as an essential nitrate signaling component (Xu et al., 2016). It regulates and works upstream of *AtNRT1.1*. *AtLBD37/38/39* repressed the expression of genes involved in nitrate uptake and assimilation to respond to nitrate status (Rubin et al., 2009). *AtSPL9, AtNAC4, AtTGA1*, and *AtTGA4* have been identified by using system approaches to function in regulating nitrate response (Krouk et al., 2010; Alvarez et al., 2014; Vidal et al., 2014). *AtCPK10/30/32*, Ca^2+^-depended protein kinases, were recently identified by a nitrate-sensitized and targeted functional genomic screen and play key roles in nitrate signaling (Liu K.H. et al., 2017).

Notably, *AtNLP7* has been demonstrated as a central component in regulating nitrate response in *Arabidopsis*. Nitrate induction of *NIA1, NIA2, NRT2.1*, and *NRT2.2* were all severely reduced in *nlp7* mutants (Castaings et al., 2009). The subcellular localization of AtNLP7 is regulated by nitrate: it accumulates in the nucleus when nitrate is present (Marchive et al., 2013). The nuclear retention is controlled by the phosphorylation of the conserved serine205 by *AtCPK10/30/32* in the N-terminal region of AtNLP7 (Liu K.H. et al., 2017). In addition, other members of the AtNLP family can bind the NRE and modulate nitrate responsive genes (Konishi and Yanagisawa, 2013).

Maize is an essential food and cash crop in many parts of the world. Much of the world’s nitrogen fertilizer is used to support the growth and high yield of maize. However, little is known about the regulation of nitrate assimilation in maize at the molecular and genetic levels. It has been reported that the expression levels of maize nitrate transporters *ZmNRT2.1* and *ZmNRT2.2* exhibit two distinct peaks in different growth stages under low nitrate conditions (Garnett et al., 2013). On the other hand, the expression of *ZmNRT2.1* and *ZmNRT2.2* in the roots of seedlings and adult plants are inhibited by local low nitrate while enhanced by local high nitrate (Yu et al., 2014). Additionally, most *ZmNRT2* family members are responsive to nitrogen treatments (Garnett et al., 2015). GS gene *ZmGln1-3* is localized in mesophyll cells and plays an important role in grain production (Martin et al., 2006). However, there have been no nitrate regulatory genes characterized in maize. In this study, we identified all nine *ZmNLP* genes in maize and analyzed their expression profiles. Genetic and molecular analyses suggest that *ZmNLP6* and *ZmNLP8* play essential roles in nitrate signaling and assimilation. Both genes are localized in the nucleus. Y1H assay revealed that ZmNLP6 and ZmNLP8 could bind NRE of *ZmNRT1.2* and *ZmNiR2* directly *in vitro*. Furthermore, constitutively overexpressing *ZmNLP6* and *ZmNLP8* in *Arabidopsis* can promote growth and yield under low nitrate conditions, implicating their potential in improving maize NUE.

## Materials and Methods

### The Characterization of the *NLP* Genes in Plants

To character the NLP members, the protein sequences of NLP family in *Arabidopsis* and rice were used as query sequences to perform BLASTp in 34 species in plant genomics resource^1^. The BLASTp were used with an *e*-value cutoff set to 1*e*-003. The isolated protein sequences were examined using the domain analysis program Hidden Markov Model (HMM) of SMART^2^ and Pfam^3^ with the default cutoff parameters (Bateman et al., 2002; Letunic et al., 2012). We obtained the sequences with the Pfam number PF02042.10 and PF00564.19, which contained a typical RWP-RK and PB1 domains, from the maize genome sequences using a Perl-based script.

### Phylogenetic Analysis, Gene and Protein Structure of the NLP Members

The full length NLP protein sequence of *Arabidopsis*, maize, sorghum, and rice were used for constructing phylogenetic tree with the neighbor-joining (NJ) method of the MEGA7 program^4^ using the p-distance and complete default option parameters. The reliability of the obtained trees was tested using a bootstrapping method with 1000 replicates. The images of the phylogenetic trees were drawn using MEGA7. The genomic and CDS of *NLP* members were downloaded from the plantGDB database^5^. The gene structures of *NLP* genes were generated using the GSDS^6^. The position of GAF-Like, RWP-RK and PB1 domain was indicated from the Pfam results.

### Sequence Alignment, Chromosomal Location and Characterization of NLP Members in Maize

The amino acid sequences of ZmNLPs were aligned using the ClustalX program with BLOSUM30 as the protein-weight matrix. The chromosomal location was retrieved from the maize genome data^7^, and the genes were mapped to the chromosomes using MapDraw program. The isoelectric points and molecular weights of these proteins were obtained with the help of the proteomics and sequence analysis tools on the ExPASy Proteomics Server^8^.

The conserved motif was discovered by Motif discovery in MEME^9^ using 248 NLP proteins in 34 species. The logos were created and downloaded by Multiple Em for Motif Elicitation.

### Plant Growth and Treatments

Maize B73 inbred seeds were grown in the fields every year (2014–2016) and a part of 0–20 cm underground roots and ear leaves(whole) were collected at the stage vegetative 3 (V3), vegetative 5 (V5), vegetative 9 (V9), vegetative 13 (V13), reproductive 1 (R1), and vegetative 18 (V18) (Sekhon et al., 2011). These are important stages for the growth and development of maize. A part of 0–20 cm underground roots, ear leaf (whole), ear stem (whole), ear sheath (whole), tassels (whole), and grains in reproductive 1 stage (15 days after pollination) were collected for testing the gene expression every year. Maize plants used for testing the induction of nitrate responsive genes were grown on matrix watered with B5 solution to 3-leaf stage, and then the endosperm were removed from the seedlings. The uniform seedlings were transferred to hydroponics system (Liu W. et al., 2017) with 2.5 mM ammonium succinate for 2 days, followed by the treatments with 0.2 mM or 10 mM KNO_3_/KCl for 2 h. The roots were collected for testing the expression of *ZmNLP* genes. Maize seeds were grown on matrix watered with different nitrate concentrations (0, 0.2, 2.5, 5, and 10 mM KNO_3_) for 2 weeks and then the shoots and roots were collected for detecting the expression of *ZmNLP* genes.

*Arabidopsis* plants used for testing the YFP fluorescence intensity were grown on plates with nitrate medium (10 mM KNO_3_) for 4 days and observed under a fluorescence microscope (Nikon Eclipse Ti-s). The fluorescence of roots was quantified using Image J. For *Arabidopsis* plants used for qPCR analysis of nitrate responsive genes, seedlings were grown in aseptic hydroponics (initial medium with 2.5 mM ammonium succinate) as previously described (Xu et al., 2016) for 7 days and then treated with 10 mM KNO_3_ or KCl for 2 h. RNA was extracted from the roots for testing the expression of nitrate responsive genes.

### qPCR

Total RNA was isolated using a total RNA miniprep kit (CWBIO). Template cDNA samples were prepared using the EasyScript One-Step gDNA Removal and cDNA Synthesis SuperMix kit (TRAN). qPCR reaction was prepared with UltraSYBR Mixture (With Rox I) followed by performing qPCR using QuantStudio^TM^ 6 (Thermo Fisher Scientific). *ZmActin1* (J01238), Zm*UBCE* (GRMZM2G027378) (Manoli et al., 2012), *AtTUB2* (At5g62690), and *AtActin8* (At1g49240) were used as the internal reference genes.

### Subcellular Localization Assay

The coding regions of *ZmNLP6* and *ZmNLP8* containing the stop codon were cloned into pMDC43 (Invitrogen). The pMDC43-GFP-ZmNLP6 and pMDC43-GFP-ZmNLP8 were transiently transformed into *Nicotiana benthamiana* epidermal cells and *Arabidopsis* mesophyll protoplasts. The GFP fluorescence was observed using a confocal microscope (Leica TCS SP5II).

### Yeast One-Hybrid (Y1H) Assay

Yeast one-hybrid (Y1H) assay was performed using the system as described previously (Lin et al., 2007). The full length of cDNA for *ZmNLP6* and *ZmNLP8* were ligated into the pJG4 vector and the putative NRE from the 5′ and 3′ flanking sequences of nitrate uptake and assimilation genes in maize were ligated into the pLacZ-2U vector. The constructed vectors were co-transferred into yeast EGY48 competent cells and grew the cells on SD/-Trp/-Ura medium for 4 days, and then positive clones were transferred into a new plate of SD/-Trp/-Ura with X-gal.

### Molecular Constructs and Transactivation Activity Assay

The *nlp7-4* mutant was isolated from EMS-treated seeds of transgenic lines containing a nitrate-regulated promoter fused to a yellow fluorescent protein (NRP::YFP) (Wang et al., 2009). *ZmNLP6* and *ZmNLP8* were amplified from the B73 inbred line by PCR. The constructs p35S::ZmNLP6 and p35S::ZmNLP8 in binary vector pSuper1300 were transformed into the *nlp7-4* mutant (Xu et al., 2016) using the Agrobacterium-mediated floral dip method. Homozygous transgenic lines were isolated and two of these lines for each *ZmNLP* gene were selected for further investigation. All primers were listed in Supplementary Table S1.

### Nitrate Metabolite Measurement

For nitrate content detection, seedlings of WT, *nlp7-4*, and transgenic lines were grown in 1/2 MS medium (pH = 5.7, 10 mM KNO_3_ and 10 mM NH_4_NO_3_) for 7 days and then collected 0.05–0.1 g of the whole seedlings. The samples were milled into powder using a RETCH MM400 and then added into l mL ddH_2_O followed by boiling at 100 °C for 30 min. The cooled sample was centrifuged at 13000 rpm for 10 min and 400 μL supernatant was collected to the flow cell. The nitrate content was detected by hydrazine reduction method using AutoAnalyzer 3 continuous flow analytical system (SEAL Analytical).

Nitrate reductase activity was detected by sulfanilamide colorimetric method as described previously (Ferrario-Mery et al., 1998). Amino acid content in tissue was determined using ninhydrin colorimetric analysis as described previously (Rosen, 1957).

### Growth and Yield Measurement

For root architecture, WT, *nlp7-4, ZmNLP6* and *ZmNLP8* transgenic lines grown vertically on agar plates containing 0.2, 2.5, and 5 mM KNO_3,_ respectively, for 10 days. The primary root length was measured by Image J and the lateral root number was counted under a dissecting microscope (Optec). For biomass measurement, seedlings were grown on KNO_3_ solid medium 0.2, 2.5, and 5 mM KNO_3_, respectively, for 10 days and fresh weight was measured or seedlings were dried under 65°C for 3 days to obtain the dry weight.

For yield measurement, seedlings were grown in pots with matrix at 22°C, 16 h light/8 h dark photoperiod in growth room and watered with 0.2 mM or 5 mM KNO_3_. The seeds were collected when plants were mature and dried at 30°C for 2 weeks.

## Results

### *NLP* Gene Family in Maize

**FIGURE 1:**
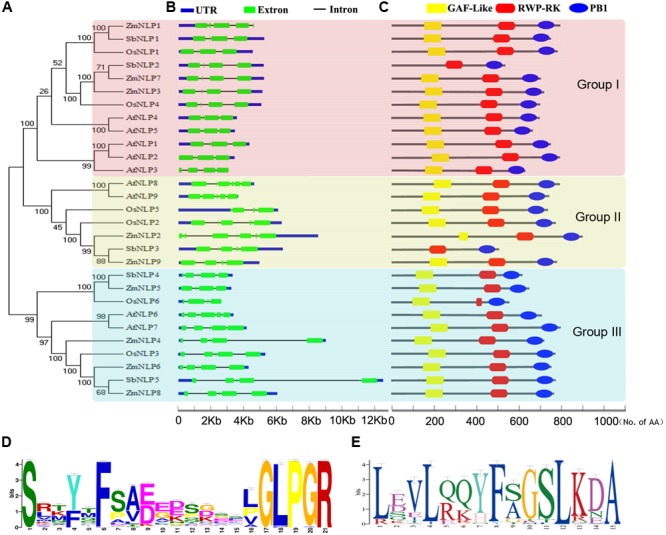
Phylogenetic analysis, schematic diagram for gene and protein structures, and conserved motifs of NLP proteins in *Arabidopsis*, maize, sorghum, and rice. **(A)** The phylogenetic analysis. The phylogenetic tree was constructed based on a complete protein sequence alignment of the NLPs by the neighbor-joining method using MEGA7 software. **(B)** The schematic diagram for exon/intron structure of the *NLP* family genes in *Arabidopsis*, maize, sorghum, and rice. The exon, intron, and UTR are represented by the green box, black line, and blue box, respectively. The scale bar represents 12 kb. **(C)** The schematic diagram for protein structure. The black lines indicate the peptide chain. The yellow squares, red cylinders, and blue ellipses represent the GAF-like, RWP-RK, and PB1 domains, respectively. The scale bar represents 100 amino acids. **(D,E)** The conserved motifs of NLP proteins in *Arabidopsis*, maize, sorghum, and rice. **(D)** The GAF motif logo; **(E)** The GSL motif logo.

To find the *NLP* homologous genes in maize, BLASTp of 34 species using NLP family amino acid sequences of *Arabidopsis* and rice as queries were performed from the plant genomics resource^10^. The search results were further verified by HMM of SMART and Pfam tools, and the proteins containing RWP-RK and PB1 domains were considered as NLP homologs. By using this strategy, 248 proteins were defined as NLP homologs in 34 species (Supplementary Figure S1). NLP proteins exist in all land plants we searched. Nine *NLP* members were found in the maize genome (**Table 1**). We named these nine members, *ZmNLP1* to *9*, mainly following their chromosomal locations except GRMZM2G475305 as *ZmNLP6* to be consistent with nomenclatures proposed in the previous study (Burdo et al., 2014) and GRMZM2G176655 on chromosome 6 alternately named *ZmNLP4*. The genes of the *ZmNLP* family are distributed over eight of the 10 maize chromosomes (except Chr.4 and Chr.9) (Supplementary Figure S2). Pairwise comparison of the ZmNLP proteins revealed levels of identity ranging from 16.67% between ZmNLP5 and ZmNLP7 to 66.08% between ZmNLP6 and ZmNLP8 (Supplementary Figure S3). Three sister pairs (*ZmNLP3/7, ZmNLP2/9*, and *ZmNLP6/8*) of paralogs with high identity (>60%) and strong bootstrap support (>90%) (**Figure 1A**) were found, implicating a paralogous pattern of *ZmNLP* divergence by gene duplication in maize. The alignment results of the nine full length ZmNLP amino acid sequences were shown in Supplementary Figure S4. Two conserved domains, RWP-RK and PB1, were found to be separated by an area of low similarity in the C-termini of ZmNLPs.

**Table 1:** The information of NLP family in maize.

Gene	Gramene ID	Location	Group	AA No.	ORF	MW	pI
ZmNLP1	GRMZM2G109509	Chr1:7081037-7085736	I	953	2859	106.23	5.64
ZmNLP2	GRMZM2G031398	Chr2:33701563-33710286	II	1089	3267	118.44	6.62
ZmNLP3	GRMZM2G048582	Chr2:198102096-198107328	I	872	2616	95.61	6.33
ZmNLP4	GRMZM2G176655	Chr6:133982025-133991228	III	873	2619	97.65	7.69
ZmNLP5	GRMZM2G042278	Chr5:76715126-76718411	III	786	2358	82.89	5.91
ZmNLP6	GRMZM2G475305	Chr3:1540716-1545106	III	916	2748	100.99	6
ZmNLP7	GRMZM2G053298	Chr7:147146774-147152106	I	851	2553	93.39	6.27
ZmNLP8	GRMZM2G375675	Chr8:29209906-29216097	III	927	2781	102.08	5.63
ZmNLP9	GRMZM2G105004	Chr10:127592227-127597271	II	1205	3615	102.83	6.06

*AA No., the number of amino acids; ORF, the length of Open Reading Frame, base pair (bp); MW, Molecular Weight, (Kilo Dolton).*

To analyze the phylogenetic relationship of NLP proteins, the unrooted phylogenetic tree was generated based on the full length amino acid sequences of NLP proteins from *Arabidopsis* (9), maize (9), sorghum (5), and rice (6) using MEGA7.0. As shown in **Figure 1A**, the NLPs were divided into 3 groups (Group I, II, and III with different colors). In each group, the NLP proteins from maize, sorghum, and rice were closely clustered. Exon/intron structures of these *NLPs*, shown in **Figure 1B**, revealed an obvious difference among groups: a module with four exons and three introns was found in most group I members with the smallest size in the second exon. The group II members mainly contain five exons and four introns, and two smaller exons separate three bigger ones. A module of five exons and four introns also found in group III members, but the third exon is the smallest one. Thus, the length and position of introns make great contributions to the diversity of gene structure in each group. For the ZmNLP proteins, the RWP-RK and PB1 domains were found to be well conserved in all tested members, and GAF-like domain were also found in the N-termini of ZmNLPs (**Figure 1C**). In addition, the results revealed two novel motifs, GAF motif and GSL motif, in the NLP family by using MEME analysis (**Figures 1D,E** and Supplementary Figure S4). The GAF motif (SX_4_FX_10_GLPGR), which is located in the N-termini of NLPs, is the main part of the GAF-Like domain and critical for nitrate signaling transduction in *Arabidopsis* (Wang et al., 2009; Liu K.H. et al., 2017). GSL motif (LX_2_LX_3_FXGSLKD) exists just in front of the RWP-PK domain and with conserved GSL residues as a core. The GSL motif was well conserved in 236 NLP proteins in 34 species, but not found in the RKD family proteins, so that it can be regarded as a marker for distinguishing the NLP proteins from RKD proteins.

### Expression Profiles of *ZmNLP* Genes

**FIGURE 2:**
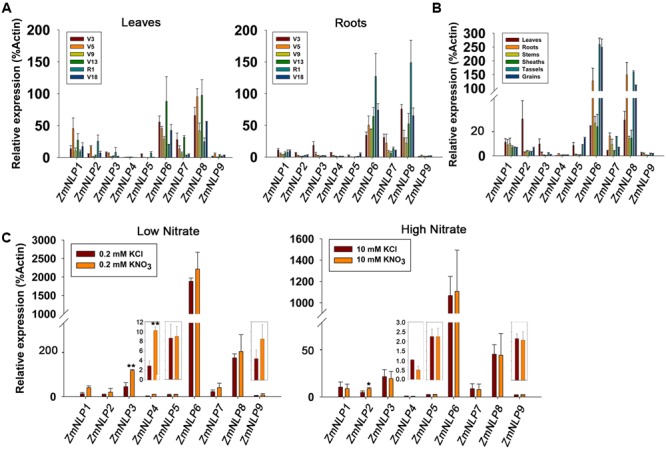
The temporal, spatial, and induction expression patterns of *ZmNLP* genes. **(A)** The temporal expression pattern of *ZmNLP*s in leaves and roots. The expanded leaves and roots were sampled at different developmental stages including vegetative 3 (V3), vegetative 5 (V5), vegetative 9 (V9), vegetative 13 (V13), reproductive 1 (R1), and vegetative 18 (V18) stages. The relative expression levels of each gene in leaves and roots were normalized to the percentage of *ZmActin1* gene. Error bars represent SD of three biological replicates. **(B)** The spatial expression pattern of *ZmNLP* genes. The expression of *ZmNLP* genes in leaves, roots, stems, sheaths, tassels, and grains was detected by qPCR. The relative expression levels of each gene in different tissues were normalized to the percentage of *ZmActin1* gene. Error bars represent SD of three biological replicates. **(C)** The relative expression of *ZmNLP* genes after low and high nitrate treatments. The 2-week-old seedlings grown under normal conditions were transferred into the medium with 2.5 mM ammonium succinate for 2 days, and then treated with 0.2 or 10 mM KNO_3_ for 2 h. KCl treatments were used as a control. The roots were collected for testing the expression of *ZmNLP* genes. The relative expression levels of each gene were normalized to the percentage of *ZmActin1* gene. Error bars represent SD of four biological replicates, (^∗∗^*P* < 0.01, ^∗^*P* < 0.05, *u*-test).

To better understand the function of each gene in the *ZmNLP* family, we examined their temporal and spatial expressions in maize B73 inbred lines by using qPCR. The results showed that different *ZmNLP* genes exhibited various expression patterns (**Figures 2A,B**). Among these genes, *ZmNLP6* and *ZmNLP8* showed significant higher expression levels in almost all tested stages and tissues, while the expression levels of *ZmNLP1* and *ZmNLP7* were much lower. *ZmNLP4* and *ZmNL9* exhibited the lowest expression levels in diverse organs and stages. Remarkably, the expression of *ZmNLP6* and *ZmNLP8* was the highest in the vegetative 13 (V13) stage in leaves and reproductive 1 (R1) stage in roots. V13 is the stage of tasseling and R1 is grouting stage, both of which are essential for maize production, suggesting that *ZmNLP6* and *ZmNLP8* may play outsized roles in these stages critical to maximizing maize yield. The expression of *ZmNLP6* and *ZmNLP8* was higher in roots, tassels, and grains, while *ZmNLP2* and *ZmNLP3* was higher in leaves than in other organs (**Figure 2B**). The expression of *ZmNLP1* was relatively uniform, while *ZmNLP7* was more highly expressed in both roots and tassels. The expression of *ZmNLP5* was relatively low, mainly expressed in leaves, tassels, and grains. We also used the second housekeeping gene *ZmUBCE* for testing the expression of these genes and similar results were found (Supplementary Figure S5). These results suggest that *ZmNLP6* and *ZmNLP8* may play more important roles in different developmental stages and tissues compared with the other members of *ZmNLPs* in maize.

To test if *ZmNLPs* are responsive to different nitrate conditions, we investigated the expression of all *ZmNLP* genes in roots. Seedlings were grown on 1/2MS medium (10 mM KNO_3_ and 10 mM NH_4_NO_3_) for 2 weeks, and then transferred into 2.5 mM ammonium succinate for 2 days followed by treatments with 0.2 or 10 mM KNO_3_ for 2 h. As shown in the **Figure 2C**, *ZmNLP3* and *ZmNLP4* were induced by 0.2 mM KNO_3_ in roots. After 10 mM KNO_3_ treatment, the expression of most *ZmNLPs* was not induced except *ZmNLP2* (**Figure 2C** and Supplementary Figure S5). Thus, most *ZmNLP* genes were not induced by nitrate.

**FIGURE 3:**
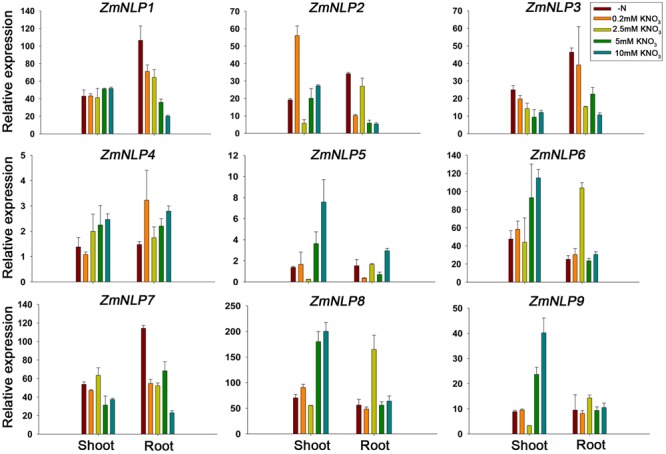
Expression patterns of *ZmNLP* genes under different nitrate conditions. The maize seedlings were grown on matrix for 2 weeks and watered with media containing 0, 0.2, 2.5, 5, or 10 mM KNO_3_. The shoots and roots were collected for detecting the expression levels of each *ZmNLP*. The relative expression levels of each gene in shoots and roots were normalized to the percentage of *ZmActin1* gene. Error bars represent SD of three biological replicates.

To test the expression patterns of *ZmNLP* genes under various nitrate conditions, 2-week-old seedlings were grown under 0, 0.2, 2.5, 5, and 10 mM KNO_3_ conditions and RNAs were extracted from the shoots and roots for qPCR analysis. The results showed that the expression levels of the *ZmNLP4, ZmNLP6*, and *ZmNP8* were generally increased with the higher concentrations of nitrate in shoots, while the highest peak was observed at 2.5 mM nitrate concentration for *ZmNLP6* and *ZmNLP8* in the roots (**Figure 3**). However, the expression of *ZmNLP3* and *ZmNLP7* generally decreased in both shoots and roots with the increase of nitrate concentrations. For *ZmNLP1*, the expression was stable in shoots while decreasing with increased nitrate concentrations (**Figure 3**). These data indicate that different *ZmNLPs* exhibit various expression profiles. The expression of *ZmNLP6* and *ZmNLP8* in shoots was the highest and increased with increasing nitrate concentrations, indicating that both genes are the strongest candidates for playing important roles in regulating nitrate metabolism in maize. The second housekeeping gene *ZmUBCE* were used for testing the expression of these genes and the results were similar to those obtained by *ZmActin1* (Supplementary Figure S6). Therefore, we focused on *ZmNLP6* and *ZmNLP8* in the following studies.

### Cloning and Functional Identification of *ZmNLP* Genes in Nitrate Signaling

**FIGURE 4:**
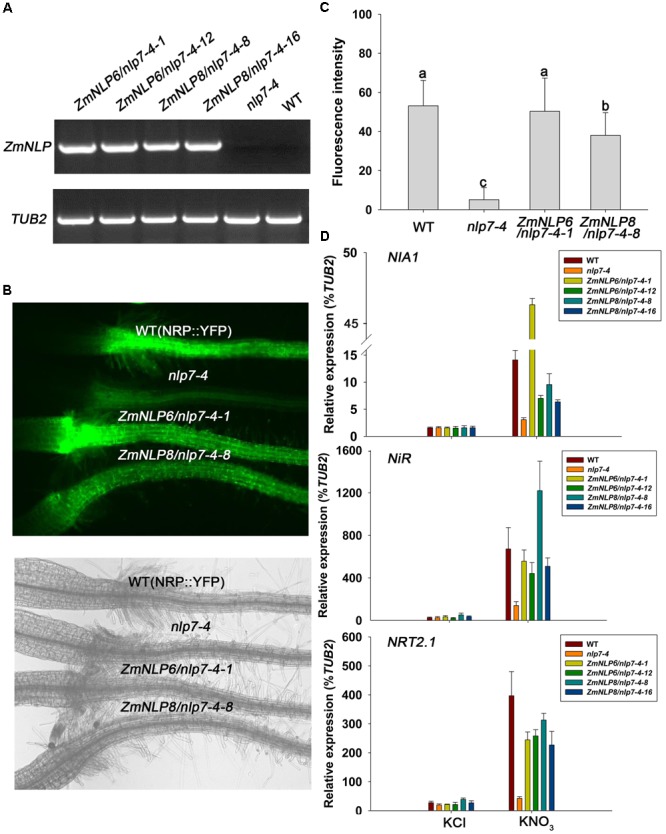
*ZmNLP6* and *ZmNLP8* modulate nitrate signaling. **(A)** The transcript expression of *ZmNLP6* and *ZmNLP8* in WT, *nlp7-4*, and transgenic lines by RT-PCR. *Arabidopsis* plants were grown on 1/2 MS medium (pH = 5.7) for 7 days and then the whole seedlings were collected for RNA extraction. The primers qZmNLP6-F, qZmNLP8-F, and 1300RT-R were used for confirming WT and the transgenic lines. *TUB2* was used as a reference gene. **(B)** Root fluorescence phenotypes of *ZmNLP6* and *ZmNLP8* transgenic lines. The seedlings were grown on 10 mM KNO_3_ medium for 4 days. Fluorescence and visible light images were captured with a fluorescent microscope. **(C)** Quantification of root fluorescence of WT, *nlp7-4*, and transgenic lines. The plants were grown on the same conditions as **(B)**. Error bars represent SD (*n* = 60). Different letters indicate statistically significant difference (*P* < 0.05, *t*-test). **(D)** The expression of nitrate responsive genes in WT, *nlp7-4*, and *ZmNLP6/nlp7-4* and *ZmNLP8/nlp7-4* transgenic lines after nitrate treatment. The seedlings grown on medium with 2.5 mM ammonium succinate for 7 days were treated with 10 mM KNO_3_ for 2 h. 10 mM KCl treatment was used as a control. The roots were collected for testing the expression of nitrate responsive genes. The relative expression levels of each gene were normalized to the percentage of *TUB2* gene. Error bars represent SD of five biological replicates.

AtNLP7 has been reported as a key regulator in nitrate signaling (Marchive et al., 2013). The *Arabidopsis* mutant *nlp7-4* with a mutation (C to T) at the position 62, converting Gln to a stop codon, was identified by a forward genetic screen (Xu et al., 2016). The *nlp7-4* plants contain NRP::YFP (a nitrate-regulated promoter fused to a yellow fluorescent protein) construct and are defective in nitrate regulation of nitrate responsive genes. To investigate if *ZmNLP6* and *ZmNLP8* are involved in nitrate signaling, we cloned them from the cDNAs of B73 inbred line and overexpressed them in the mutant *nlp7-4*, respectively, using a 35S promoter. Transgenic plants overexpressing the *ZmNLP6* and *ZmNLP8* were generated and two independent homozygous lines (*ZmNLP6/nlp7-4-1* and *ZmNLP6/nlp7-4-12* for *ZmNLP6* transgenic lines, *ZmNLP8/nlp7-4-8* and *ZmNLP8/nlp7-4-16* for *ZmNLP8* transgenic lines) were selected for following investigation (**Figure 4A**). Nitrate-responsive expression was monitored using a construct containing the YFP fused to a nitrate-responsive synthetic promoter (NRP) (Wang et al., 2009).

The transgenic lines were grown on nitrate medium and YFP fluorescence of these lines was found to be partially or completely restored to WT levels (**Figures 4B,C**), indicating that the *ZmNLP6* and *ZmNLP8* may function in nitrate signaling. Furthermore, we investigated the expression of endogenous nitrate-responsive genes including *NIA1, NiR*, and *NRT2.1* and found that the induction levels of all three genes were recovered in *ZmNLP6* and *ZmNLP8* transgenic lines after nitrate treatments (**Figure 4D**). The expressions of these genes were also normalized to the percentage of second housekeeping gene *AtActin8* and similar results were found (Supplementary Figure S7). These results suggest that *ZmNLP6* and *ZmNLP8* can complement the *Atnlp7* mutant and thus play important roles in nitrate signaling.

### Subcellular Localization of ZmNLP6 and ZmNLP8

**FIGURE 5:**
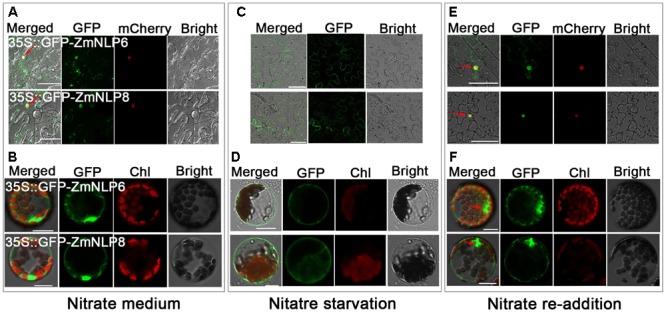
ZmNLP6 and ZmNLP8 are localized in the nucleus and are regulated by nitrate. **(A,C,E)** The subcellular localization of ZmNLP6 and ZmNLP8 in *Nicotiana benthamiana* epidermal cells. *Nicotiana benthamiana* seedlings were grown on 5 mM KNO_3_ for 5 weeks **(A)** and then treated with 2.5 mM ammonium succinate for 4 days **(C)** followed with 5 mM KNO_3_ for 12 h **(E)**. The constructs pMDC43-ZmNLP6 and pMDC43-ZmNLP8 were transiently co-transformed into *Nicotiana benthamiana* epidermal cells with the *mCherry* construct, respectively. Red mCherry fused with H2B native promoter and H2B protein was used as a marker for nucleus localization. The scale bar represents 50 μm. GFP fluorescence in the nucleus was observed and marked by red arrows. **(B,D,F)** The subcellular localization of ZmNLP6 and ZmNLP8 in *Arabidopsis* mesophyll protoplasts. The *Arabidopsis* WT seedlings were grown on 5 mM KNO_3_ for 4 weeks **(B)** and then treated with 2.5 mM ammonium succinate for 6 days **(D)** followed with 5 mM KNO_3_ for 12 h **(F)**. The constructs pMDC43-ZmNLP6 and pMDC43-ZmNLP8 were transiently into *Arabidopsis* mesophyll protoplasts. The scale bar represents 20 μm. GFP fluorescence in the nucleus was observed and marked by red arrows.

To investigate the subcellular localization of ZmNLP6 and ZmNLP8 proteins, the full length CDS of the two genes driven by 35S promoter were fused with GFP in N-terminal. The 35S::GFP-ZmNLP6 and 35S::GFP-ZmNLP8 constructs were transiently transformed into *Nicotiana benthamiana* epidermal cells and *Arabidopsis* mesophyll protoplasts, the results showed that both ZmNLP6 and ZmNLP8 were mainly localized in the nucleus and slightly in cytosol (**Figures 5A,B**). As AtNLP7 has been reported to be regulated by nitrate via a nuclear retention mechanism (Marchive et al., 2013), we next tested the subcellular localization of ZmNLP6 and ZmNLP8 proteins under nitrate starvation and nitrate re-addition conditions. Both ZmNLP6 and ZmNLP8 were found to be localized in cytosol after nitrate starvation (**Figures 5C,D**) while in the nucleus when nitrate was resupplied (**Figures 5E,F**). Thus, ZmNLP6 and ZmNLP8 are mainly localized in the nucleus and slightly in cytosol in the presence of nitrate while both proteins are localized in cytosol when nitrate is absent.

### *ZmNLP6* and *ZmNLP8* Regulate Nitrate Assimilation When Overexpressed in *Arabidopsis*

**FIGURE 6:**
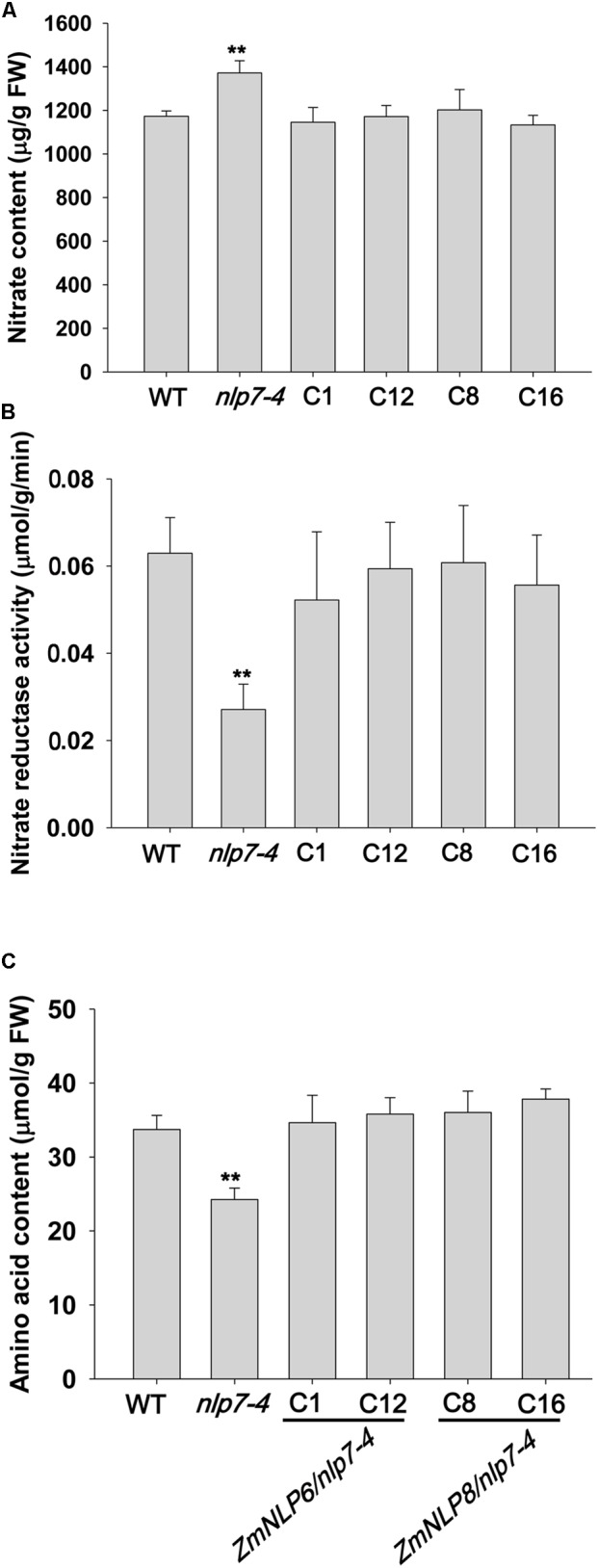
*ZmNLP6* and *ZmNLP8* affect nitrate accumulation and assimilation. **(A)** Nitrate content in seedlings. **(B)** Nitrate reductase activity in seedlings. **(C)** Amino acid content in seedlings. Seedlings of WT, *nlp7-4*, and *ZmNLP6/nlp7-4* and *ZmNLP8/nlp7-4* transgenic lines were grown on 1/2 MS medium (pH = 5.7) for 7 days and collected for testing nitrate content, nitrate reductase activity, and amino acid content. Error bars represent SD of six biological replicates (^∗∗^*P* < 0.01, *u*-test).

Previous studies have shown that nitrate assimilation is impaired and the nitrate content is increased in *nlp7* mutants (Castaings et al., 2009). To test if *ZmNLP6* and *ZmNLP8* affect nitrate assimilation, we measured nitrate content in transgenic lines and found that the increased nitrate content in *nlp7-4* was recovered to WT levels (**Figure 6A**). To investigate if this recovery is associated with nitrate reduction, we examined the NR activity and found that this activity was restored in the transgenic lines (**Figure 6B**). Furthermore, the deficiency of amino acid in *nlp7-4* mutant was completely rescued when *ZmNLP6* and *ZmNLP8* were overexpressed in *nlp7-4* mutant (**Figure 6C**). Taken together, these data indicate that *ZmNLP6* and *ZmNLP8* are involved in regulating nitrate assimilation when overexpressed in *Arabidopsis*.

In order to further explore the underling mechanism whereby *ZmNLP6* and *ZmNLP8* affect nitrate content, we examined nitrate accumulation and the expression of several genes involved in nitrate assimilation. Plants were grown on 2.5 mM ammonium succinate for 7 days and then treated with 5 mM KNO_3_ for 0, 0.25, 0.5, 1, 2, and 4 h in the presence of 2.5 mM ammonium succinate. The nitrate content in whole seedlings was investigated and the results showed that no difference was found in nitrate accumulation among the WT, *nlp7-4*, and transgenic lines (Supplementary Figure S8). However, the expression of *Gln1.1, Gln1.3, NIA2*, and *NiR* in transgenic lines was recovered to the levels even higher than in WT (Supplementary Figure S9). These findings suggest that *ZmNLP6* and *ZmNLP8* can regulate the nitrate assimilation more strongly than nitrate accumulation.

### ZmNLP6 and ZmNLP8 Can Bind NRE of *ZmNRT1.2* and *ZmNiR2* Directly *In Vitro*

**FIGURE 7:**
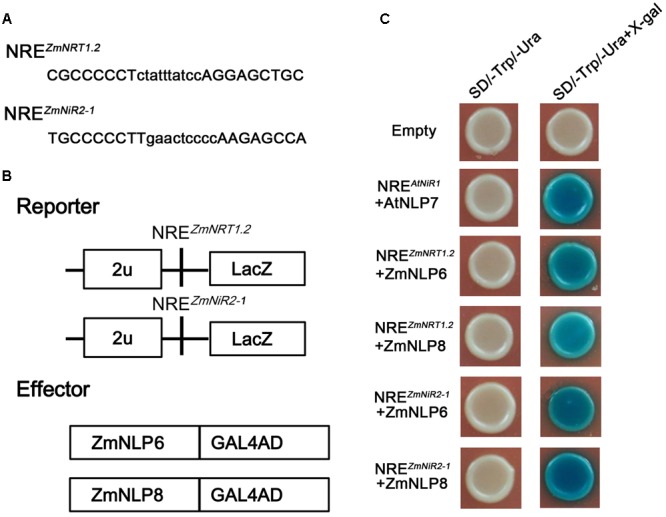
ZmNLP6 and ZmNLP8 can bind NRE of *ZmNRT1.2* and *ZmNiR2 in vitro*. **(A)** NRE sequences and **(B)** the reporters and effectors used in Y1H assay. **(C)** Y1H assay. LexA-ZmNLP6 and LexA-ZmNLP8 fusion proteins activate the expression of *LacZ* reporter gene driven by the NRE of *ZmNRT2.1* and *ZmNiR2*, respectively, in yeast. The empty vector was used as a negative control and the NRE^AtNiR1^+AtNLP7 was used as a positive control.

It has been found that *Arabidopsis* NLP proteins can bind nitrate regulatory elements (NREs) to regulate the nitrate responsive genes (Konishi and Yanagisawa, 2013). To investigate whether ZmNLP6 and ZmNLP8 can bind NREs in maize, Y1H assay was performed. Firstly, we searched for NREs with a module (tGACcCTTN_10_AAGagtcc) from the 5′ and 3′ franking sequences of nitrate uptake and assimilation genes. Nine putative NREs were obtained as shown in Supplementary Table S2. Then, the candidate NREs were used for testing the binding activity of ZmNLP6 and ZmNLP8. The results showed that ZmNLP6 and ZmNLP8 could bind the NRE-like motifs of *ZmNRT1.2* (3′UTR, with position 7299–7326) and *ZmNiR2* (promoter, -149-124), respectively (**Figures 7A–C**). But no binding activity was found between ZmNLP6/ZmNLP8 and other NREs (Supplemental Figure S10). These data suggest that the ZmNLP6 and ZmNLP8 proteins can bind putative NREs similar to what was reported for *Arabidopsis*.

### Overexpression of *ZmNLP6* and *ZmNLP8* Can Alter Root Architecture and Improve Nitrogen Use Efficiency (NUE)

**FIGURE 8:**
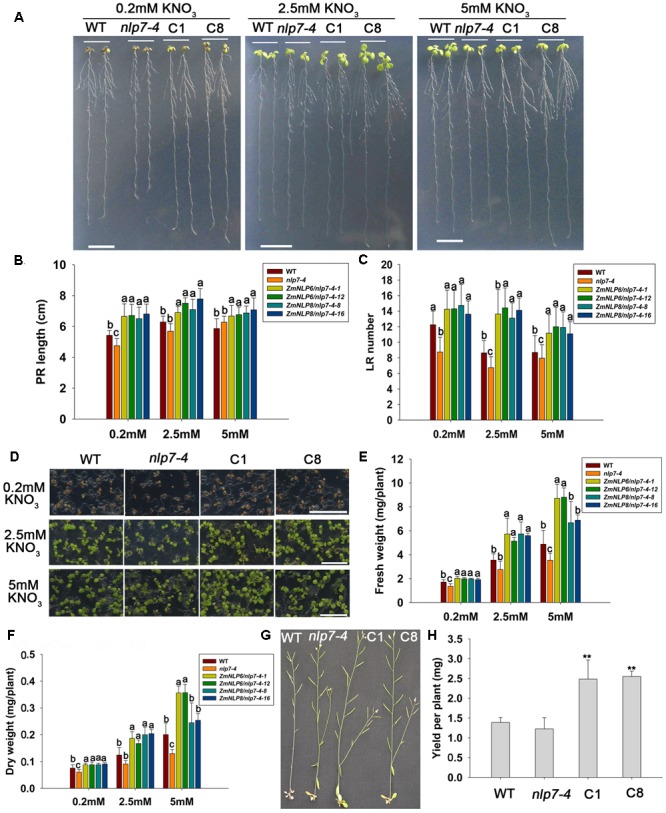
*ZmNLP6* and *ZmNLP8* can improve plant biomass under both high and low nitrate conditions and promote the seed yield. **(A)** Seedlings grown vertically on the media with 0.2, 2.5, and 5 mM KNO_3_ for 10 days, respectively. The scale bar represents 1 cm. C1 represents *ZmNLP6/nlp7-4-1* transgenic line and C8 represents *ZmNLP8/nlp7-4-8* transgenic line. **(B)** Primary root length and **(C)** lateral root number of the plants. Error bars represent SD of four biological replicates and each replicate contains 15 plants. Different letters indicate statistically significant difference (*P* < 0.05, *u*-test). **(D)** Seedlings grown horizontally on the media with 0.2, 2.5, and 5 mM KNO_3_ for 10 days, respectively. The scale bar represents 1 cm. **(E)** Fresh weigh and **(F)** dry weight of the plants. Error bars represent SD of four biological replicates and each replicate contains 40 plants. Different letters indicate statistically significant difference (*P* < 0.05, *t*-test). **(G)** WT, *nlp7-4*, and transgenic plants grown in vermiculite watered with 0.2 mM KNO_3_. **(H)** The yield per plant in WT, *nlp7-4*, and transgenic lines. Error bars represent SD of five biological replicates and each replicate contains 9 plants (^∗∗^*P* < 0.01, *u*-test).

Low NUE in agriculture system is a global problem and therefore we assessed the potential of both genes to affect root architecture and NUE of in *Arabidopsis*. We first examined the primary root length and lateral root number in plants grown vertically on the media with different nitrate concentrations (0.2, 2.5, and 5 mM KNO_3_). The results showed that the length of primary roots and number of lateral roots were higher in *ZmNLP6/nlp7-4* and *ZmNLP8/nlp7-4* transgenic lines than in WT and *nlp7-4* mutant under these three nitrate conditions (**Figures 8A–C**). To determine whether *ZmNLP6* and *ZmNLP8* can enhance NUE in plants, we investigated the biomass of WT, *nlp7-4*, and *ZmNLP6/nlp7-4* and *ZmNLP8/nlp7-4* transgenic lines under different nitrate concentrations. The results showed that the transgenic seedlings grew bigger than WT and *nlp7-4* and the biomass of the whole seedlings increased by 15, 35, and 40% more than WT under 0.2, 2.5, and 5 mM KNO_3_ conditions (**Figures 8D–F**), indicating that the *ZmNLP6* and *ZmNLP8* can rescue the deficient growth phenotype of *nlp7-4* mutant and promote plant growth under both low and high nitrate conditions.

Seed yield is an important trait for agricultural production and also for assessing the NUE of plants. Thus, we investigated the seed yield of *ZmNLP6/nlp7-4* and *ZmNLP8/nlp7-4* transgenic lines grown under both high and low nitrate conditions. The results showed that the yield per plant was higher in *ZmNLP6/nlp7-4* and *ZmNLP8/nlp7-4* transgenic lines by 44 and 45%, respectively, than in WT under low nitrate conditions (**Figures 8G,H**). However, no significant difference was found between the transgenic lines and WT when grown under high nitrate conditions (Supplementary Figure S11). These data suggest that *ZmNLP6* and *ZmNLP8* may improve plant NUE under low nitrate conditions.

## Discussion

It has been reported that *Arabidopsis NLP* genes are involved in nitrate regulation, but the functions of maize *NLP* genes remain unknown. As maize is one of the main crops of the world, identifying the genes associated with nitrate signaling and deciphering the corresponding gene networks are of great importance for improving NUE and reducing environmental pollution. In this study, we identified nine *ZmNLP* genes containing RWP-RK and PB1 domains by genome-wide analysis in maize. The *RWP-RK* superfamily includes *NLP* and *RKD* families, both of which contain RWP-RK domain (Schauser et al., 2005). *NLP* family is conserved in the land plants we searched, especially in maize (9 members), sorghum (5 members), rice (6 members), and *Arabidopsis* (9 members) (Chardin et al., 2014). This family can be divided into three subfamilies and each subfamily shows different gene structure characteristics from each other (**Figure 1A**). A previous study also reported a similar subfamily division in *Arabidopsis*, rice, and *Lotus japonicus* (Schauser et al., 2005). We found two novel NLP motifs among these searched 34 species: GAF motif (SX_4_FX_10_GLPGR) and GSL motif (LX_2_LX_3_ FXGSLKD) (**Figures 1D,E**). The GAF motif exists in the N-terminus of NLP protein, a region involved in receiving the nitrate signal (Konishi and Yanagisawa, 2013). The most conserved signature structure in GAF motif is the first serine, the fifth phenylalanine, and GLPGR. The effects of serine and proline in GAF motif for nitrate signaling transduction have been reported (Wang et al., 2009; Liu K.H. et al., 2017). The GSL motif, located in the front of the RWP-RK domain, is conserved only in the NLP proteins but not in RDK proteins.

The expression profiles of *ZmNLP* genes showed that *ZmNLP6* and *ZmNLP8* exhibited the highest expression levels among the whole gene family, especially in R1 in roots and V13 in leaves (**Figure 2A**). As roots of R1 and the leaves of V13 are important for absorbing and remobilizing nitrate to pool organs and critical for yield of maize (Hirel et al., 2001), *ZmNLP6* and *ZmNLP8* may be important for nutrient uptake and translocation. The nitrate induction was tested for all *ZmNLP* genes and the results showed that only the expression of *ZmNLP3* and *ZmNLP4* were induced by low nitrate, and induced poorly or not at all by high nitrate (**Figure 2C**), implying that a post-transcriptional regulation response to nitrate may exist. Under different nitrate conditions, *ZmNLP1, ZmNLP2, ZmNLP3*, and *ZmNLP7* exhibited relatively higher expression in roots under nitrogen starvation and they mainly belongs to ZmNLP Group I, implying that the function of Group I may be involved in nitrogen starvation (**Figure 3**). Moreover, *ZmNLP6* and *ZmNLP8* showed the highest expression levels under higher concentrations of nitrate (5 and 10 mM) in shoots while the highest levels on 2.5 mM nitrate condition in roots (**Figure 3**) and may participated in nitrate absorb and allocation. The expression patterns of *ZmNLPs* implicate that functional redundancy of *ZmNLP* family members in nitrate regulation may exist, and these *NLP* genes may play important roles in nitrate regulation in different stages and organs under various nitrate conditions.

In *Arabidopsis*, several nitrate regulatory genes have been identified and these genes can modulate genes involved in nitrate transport, assimilation, and response. But so far, no nitrate regulators have been reported in maize. We investigated the function of maize *NLP* genes and found that overexpression of *ZmNLP6* and *ZmNLP8* in *Arabidopsis nlp7-4* mutant could recover the YFP fluorescence from the NRP-YFP transgene product and the induction of nitrate responsive genes to WT levels (**Figure 4**), indicating that *ZmNLP6* and *ZmNLP8* can restore the nitrate signaling in *nlp7-4* mutant. Previous studies have reported that NRE works as an important nitrate-responsive *cis*-acting element and can be bound by NLPs in *Arabidopsis* (Konishi and Yanagisawa, 2013). Our Y1H results showed that ZmNLP6 and ZmNLP8 proteins can bind potential NREs of *ZmNRT1.2* and *ZmNiR2 in vitro* (**Figure 7**), suggesting a direct regulation of *ZmNRT1.2* and *ZmNiR2* by ZmNLP6 and ZmNLP8 may exist in maize. In *Arabidopsis, NLP7* has a profound influence on nitrate responsive genes at the transcriptional level and some target genes of NLP7 are regulated by binding to their NREs (Konishi and Yanagisawa, 2013; Guan et al., 2017; Sato et al., 2017). Thus, the ZmNLPs may bind NRE to regulate nitrate signaling in maize similar to that in *Arabidopsis*, and this regulation mechanism may be conserved in monocots and dicots. The subcellular localization of ZmNLP6 and ZmNLP8 is regulated by nitrate (**Figure 5**), similar to a mechanism controlling AtNLP7 localization in *Arabidopsis*. Our physiological and molecular analyses further revealed that the nitrate reduction process could be recovered in the *ZmNLP6/nlp7-4* and *ZmNLP8/nlp7-4* transgenic lines, indicating that both *ZmNLP6* and *ZmNLP8* can modulate nitrate assimilation when constitutively overexpressed in *Arabidopsis*. It has been reported that overexpression *AtNLP7* in *Arabidopsis* can increase fresh weight and modify root architecture (including the longer primary root and increased lateral roots) under low and high nitrate conditions. In our study, overexpression of *ZmNLP6* and *ZmNLP8* in *nlp7-4* mutant can also enhance the biomass and root development. However, the nitrate content, amino acid content, and NR activity were increased in *AtNLP7* overexpression lines while restored to WT levels in *ZmNLP* transgenic lines. In addition, we found an increase in seed yield in *ZmNLP6* and *ZmNLP8* transgenic *Arabidopsis* lines under low nitrate conditions. It remains to be further investigated if the seed yield can be increased in *AtNLP7* overexpression lines. Results shown in this study suggest the function of the group III NLPs in *Arabidopsis* and maize may be partially conserved in nitrate regulation.

Improving NUE of crops is of great importance for sustainable agriculture. Several nitrate-related genes have been implicating in improving NUE. *OsDEP1*, encoding a highly cysteine (Cys)-rich G protein γ subunit, has been reported to increase rice harvest index and grain yield under moderate levels of nitrogen fertilization (Sun et al., 2014). *OsNRT1.1B*-indica variation has been identified to enhance the ability of nitrate uptake and root-to-shoot transport to improve NUE in rice (Hu et al., 2015). In addition, overexpression of *OsNRT2.3b* can improve grain yield and NUE by increasing the capacity of pH-buffering and uptake of N, Fe, and P in rice (Fan et al., 2016). In *Arabidopsis*, overexpression of *AtNLP7* can improve plant growth under both nitrogen-limiting and -sufficient conditions (Yu et al., 2016). In this paper, our results showed that *ZmNLP6* and *ZmNLP8* could promote plant growth under both low and high nitrate conditions, and increase seed yield under low nitrate conditions (**Figure 8**). Therefore, both *ZmNLP6* and *ZmNLP8* genes may be of great potential in improving NUE of maize. It would be also interesting to assess the role of other NLP members in promoting NUE of maize in the near future.

## Author Contributions

YW, the corresponding author of the manuscript, designed the research and analysis of data for the work with the help of NC. YW, HC, and SQ: agreement to be accountable for all aspects of the work in ensuring that questions related to the accuracy or integrity of any part of the work are appropriately investigated and resolved. HC and SQ, designed the work, drafted the work or revised it critically for important intellectual content; Final approval of the version to be published. MS, ZL, and YY revised it critically for important intellectual content.

## Conflict of Interest Statement

The authors declare that the research was conducted in the absence of any commercial or financial relationships that could be construed as a potential conflict of interest.

## Footnotes

1 https://phytozome.jgi.doe.gov/pz/portal.html

2 http://smart.emblheidelberg.de/

3 http://pfam.xfam.org/

4 http://www.megasoftware.net/

5 http://www.plantgdb.org/

6 http://gsds.cbi.pku.edu.cn/

7 http://www.maizegdb.org/

8 http://expasy.org/

9 http://meme-suite.org/tools/meme

10 https://phytozome.jgi.doe.gov/pz/portal.html

## Abbreviations

CDS: coding sequence
GFP: green fluorescent protein
GS: glutamine synthetase
NiR: nitrite reductase
NR: nitrate reductase
NRE: nitrate-responsive *cis*-element
NUE: nitrogen use efficiency
PNR: primary nitrate response
WT: wide type
Y1H: Yeast one-hybrid
YFP: yellow fluorescent protein
