# Supplementary material for: Overexpression of the Maize ZmNLP6 and ZmNLP8 Can Complement the Arabidopsis Nitrate Regulatory Mutant nlp7 by Restoring Nitrate Signaling and Assimilation
Source: Front Plant Sci. 2017 Oct 5;8:1703. doi: 10.3389/fpls.2017.01703 (PMC5634353; doi:10.3389/fpls.2017.01703)
Supplement: Supplementary file 1 [file Data_Sheet_1.docx]

Supplementary Material

**Overexpression of the maize *ZmNLP6* and *ZmNLP8* can complement the *Arabidopsis* nitrate regulatory mutant *nlp7* by restoring nitrate signaling and assimilation**

**Huairong Cao^1,a^, Shengdong Qi^1,a^, Mengwei Sun^1^, Zehui Li^1^, Yi Yang^1^, Nigel M. Crawford^2^, Yong Wang^1*^**

*** Correspondence:** Corresponding Author: [wangyong@sdau.edu.cn](mailto:wangyong@sdau.edu.cn)

## 1. Supplementary Figures


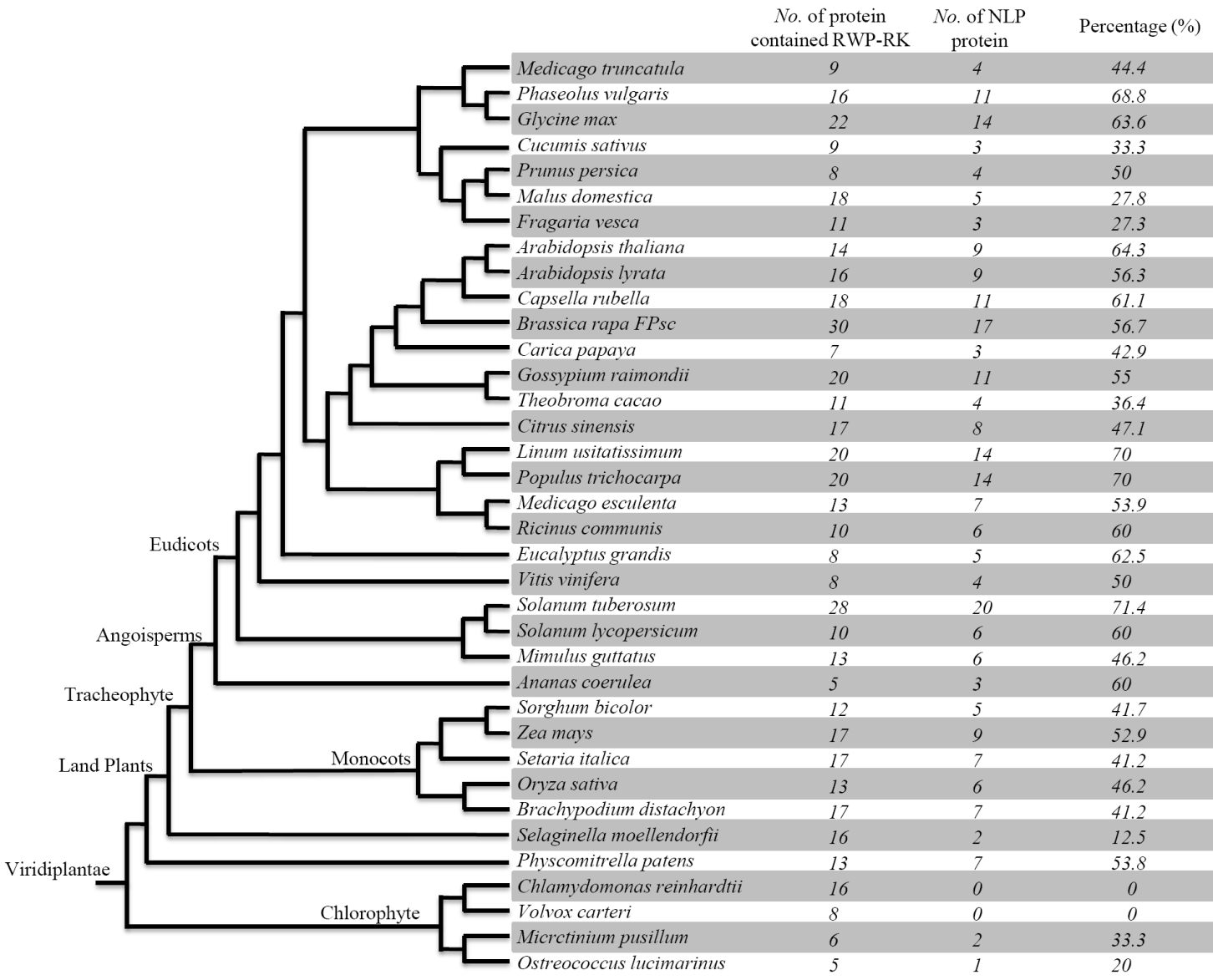
 **Supplementary Figure 1** The phylogenetic relationships of plants with completely sequenced genomes. The numbers in parentheses corresponds to the number of RWP-RK domain contained (left), NLP family (middle) and the proportion of NLP family members in RWP-RK domain contained (right).


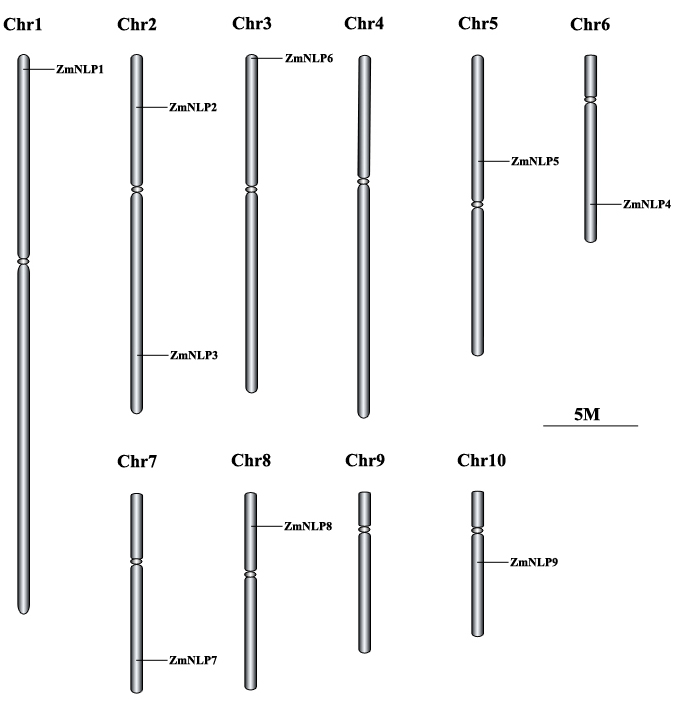


**Supplementary Figure 2** The chromosomal location of *ZmNLPs* in maize genome.


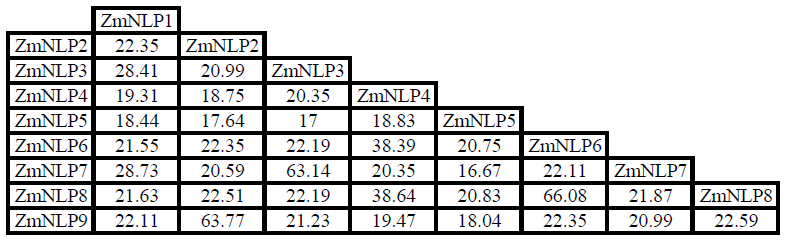


**Supplementary Figure 3** Identity matrix for NLP family in maize.


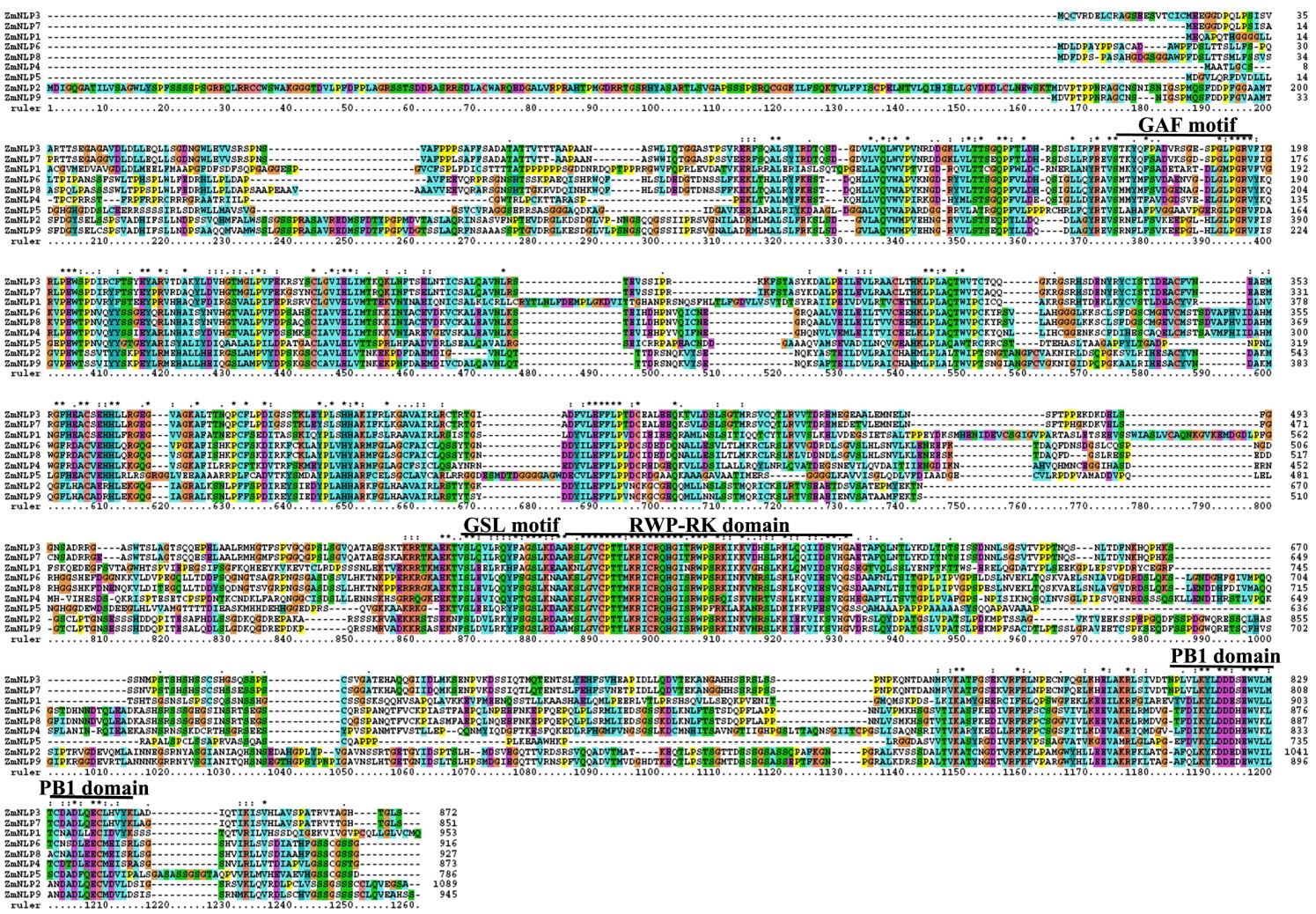


**Supplementary Figure 4** The alignment of protein sequence of ZmNLPs. The black lines indicated the conserved motifs in NLP protein.


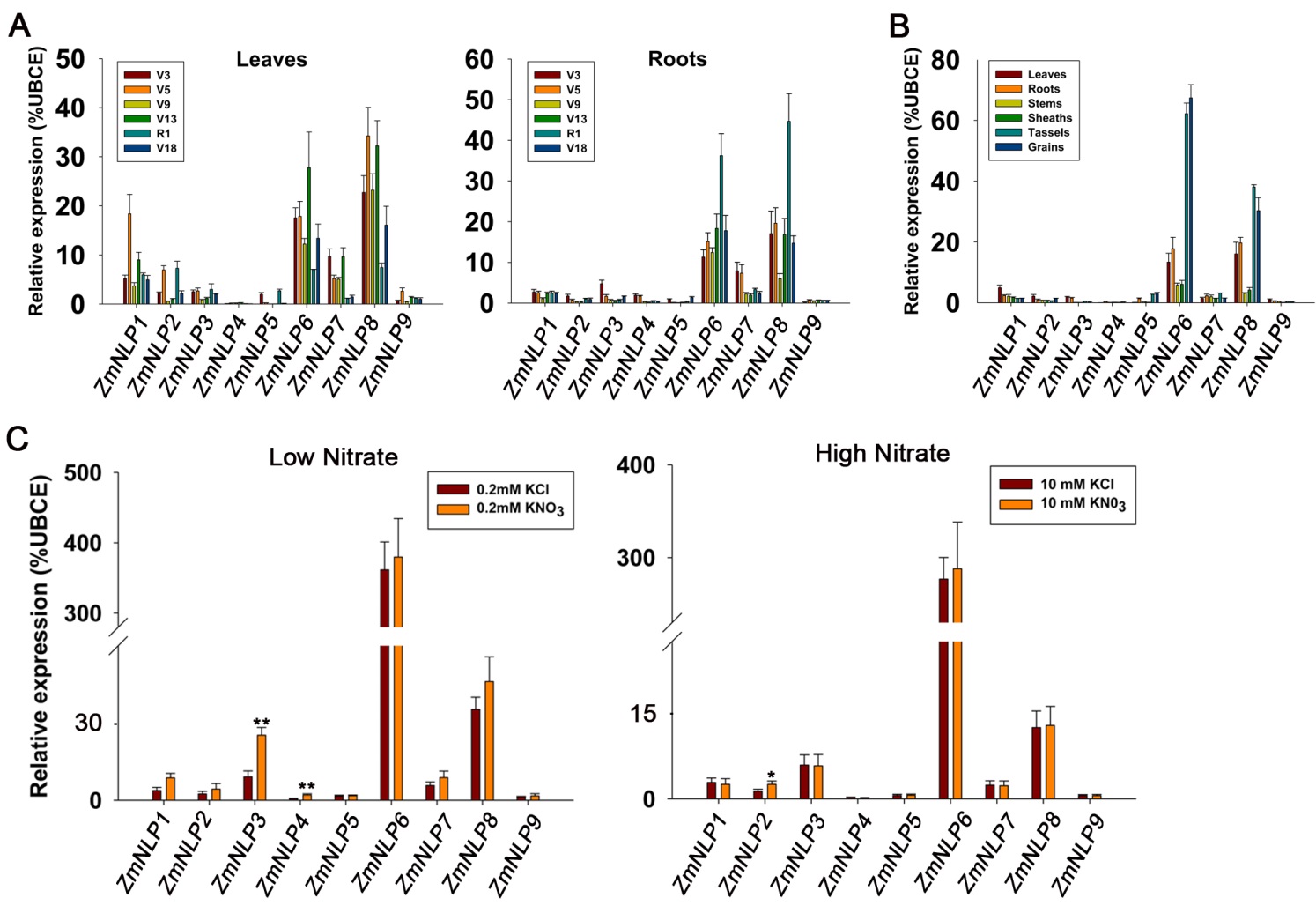


**Supplementary Figure 5** The temporal, spatial, and induction expression patterns of *ZmNLP* genes. (A) The temporal expression pattern of *ZmNLP*s in leaves and roots. The expanded leaves and roots were sampled at different developmental stages including vegetative 3 (V3), vegetative 5 (V5), vegetative 9 (V9), vegetative 13 (V13), reproductive 1 (R1), and vegetative 18 (V18) stages. The relative expression levels of each gene in leaves and roots were normalized to the percentage of *ZmUBCE* gene. Error bars represent SD of three biological replicates.

(B) The spatial expression pattern of *ZmNLP* genes. The expression of *ZmNLP* genes in leaves, roots, stems, sheaths, tassels, and grains was detected by qPCR. The relative expression levels of each gene in different tissues were normalized to the percentage of *ZmUBCE* gene. Error bars represent SD of three biological replicates.

(C) The relative expression of *ZmNLP* genes after low and high nitrate treatments. The 2-week-old seedlings grown under normal conditions were transferred into the medium with 2.5 mM ammonium succinate for 2 d, and then treated with 0.2 mM or 10 mM KNO_3_ for 2 h. KCl treatments were used as a control. The roots were collected for testing the expression of *ZmNLP* genes. The relative expression levels of each gene were normalized to the percentage of *ZmUBCE* gene. Error bars represent SD of four biological replicates, (**P<0.01,*P<0.05, u-test).


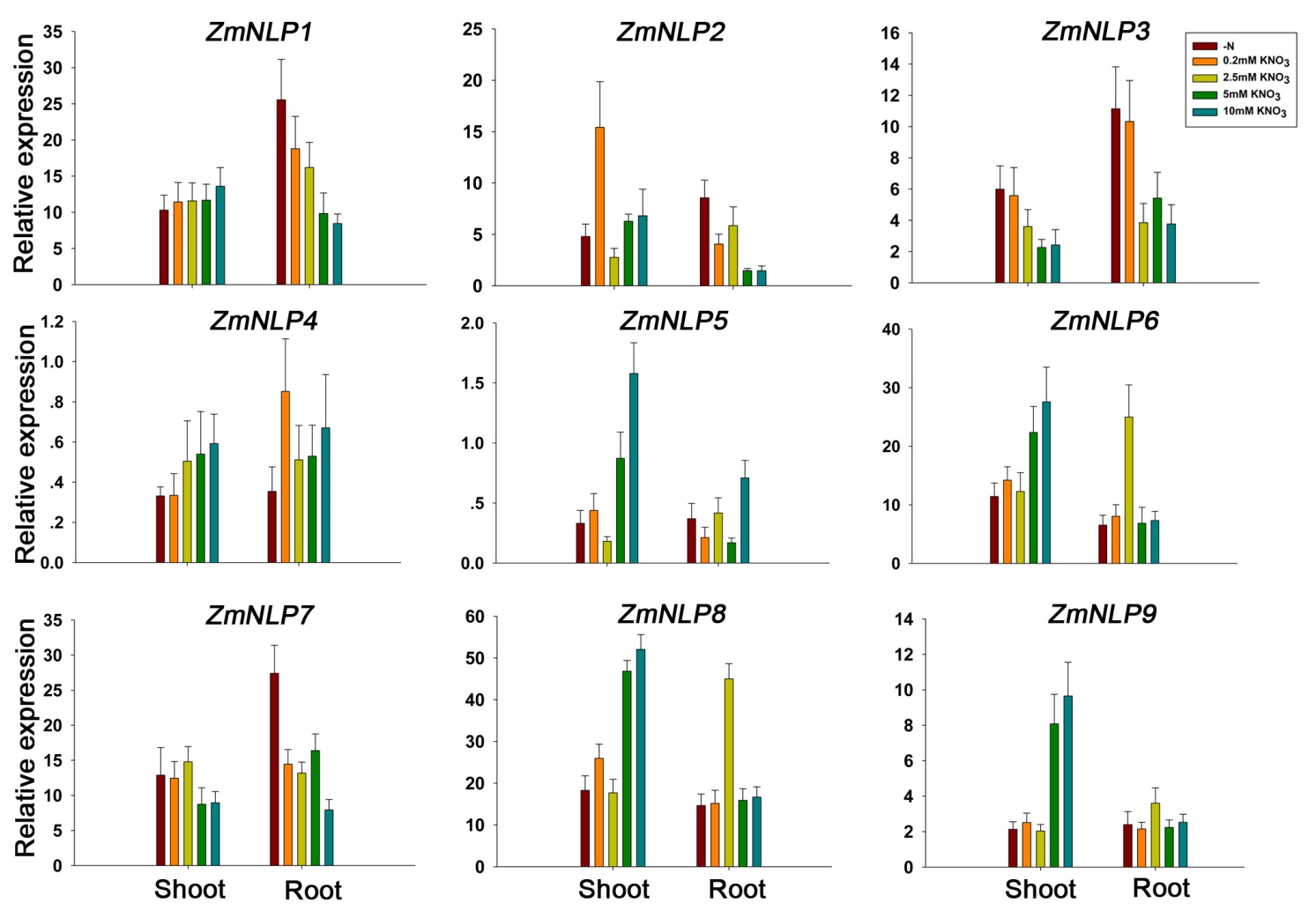


**Supplementary Figure 6** Expression patterns of *ZmNLP* genes under different nitrate conditions.

The maize seedlings were grown on matrix for 2 weeks and watered with media containing 0 mM, 0.2 mM, 2.5 mM, 5 mM, or 10 mM KNO_3_. The shoots and roots were collected for detecting the expression levels of each *ZmNLP*. The relative expression levels of each gene in shoots and roots were normalized to the percentage of *ZmUBCE* gene. Error bars represent SD of three biological replicates.


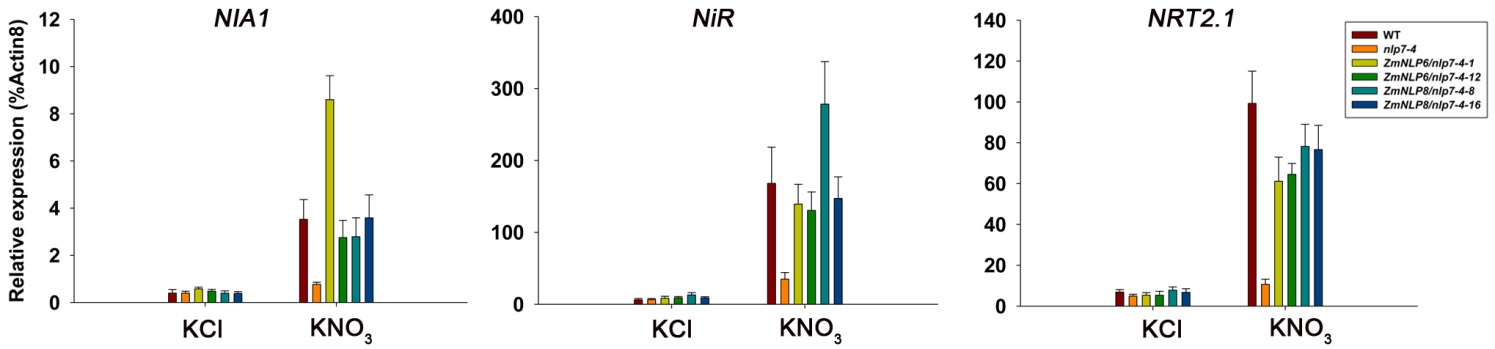


**Supplementary Figure 7** The expression of nitrate responsive genes *NIA1*, *NiR*, and *NRT2.1* in WT, *nlp7-4*, and *ZmNLP6/nlp7-4* and *ZmNLP8/nlp7-4* transgenic lines after nitrate treatment. The seedlings grown on medium with 2.5 mM ammonium succinate for 7 d were treated with 10 mM KNO_3_ for 2 h. 10 mM KCl treatment was used as a control. The roots were collected for testing the expression of nitrate responsive genes. The relative expression levels of each gene were normalized to the percentage of *Actin8* gene. Error bars represent SD of five biological replicates.

**Supplementary Figure 8** *ZmNLP6* and *ZmNLP8* did not affect nitrate uptake. Nitrate accumulation in WT, *nlp7-4*, and *ZmNLP6/nlp7-4* and *ZmNLP8/nlp7-4* transgenic lines. WT, *nlp7-4*, and transgenic lines were grown on 2.5 mM ammonium succinate for 7 d and then treated with 5 mM KNO_3_ for 0.25 h, 0.5 h, 1 h, 2 h, and 4 h. The seedlings were collected for detecting nitrate content, *chl1-5* as a positive control. Error bars represent SD of four biological replicates (*P<0.05, u-test).

*****

*****

*****

*****

*****

*****

**0.25**

**0.5**

**1**

**2**

**4**

**0**

**(h)**


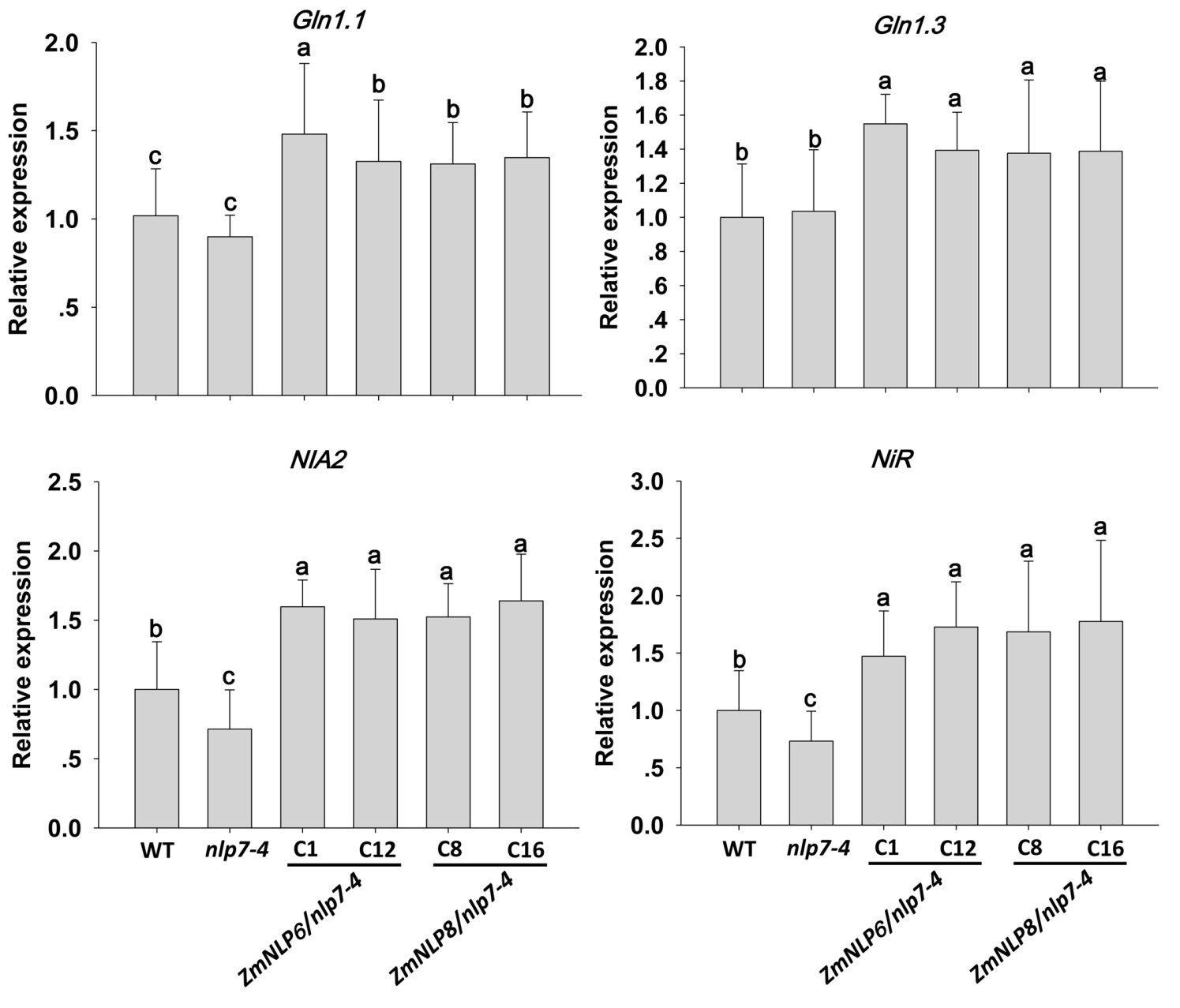


**Supplementary Figure 9** *ZmNLP6* and *ZmNLP8* were associated with nitrate assimilation. WT, *nlp7-4*, *ZmNLP6* and *ZmNLP8* transgenic lines were grown on 1/2 MS medium for 7 d and collected for RNA extract. Error bars represent SD of four biological replicates. Different letters indicate statistically significant difference (P<0.05, u-test).


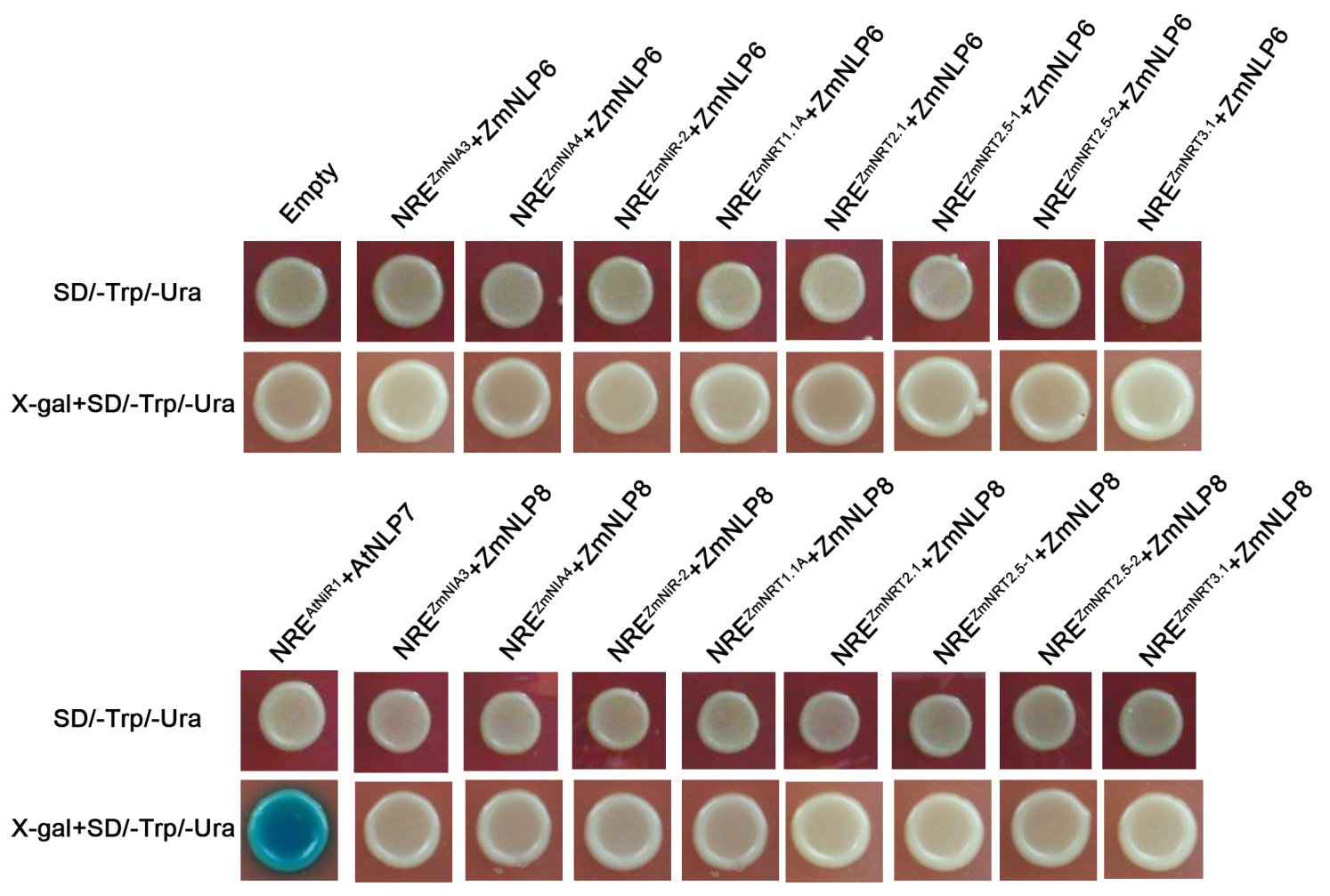


**Supplementary Figure 10** Y1H assay. The empty vector was used as a negative control, and the NRE*^AtNiR1^* and AtNLP7 as a positive control.


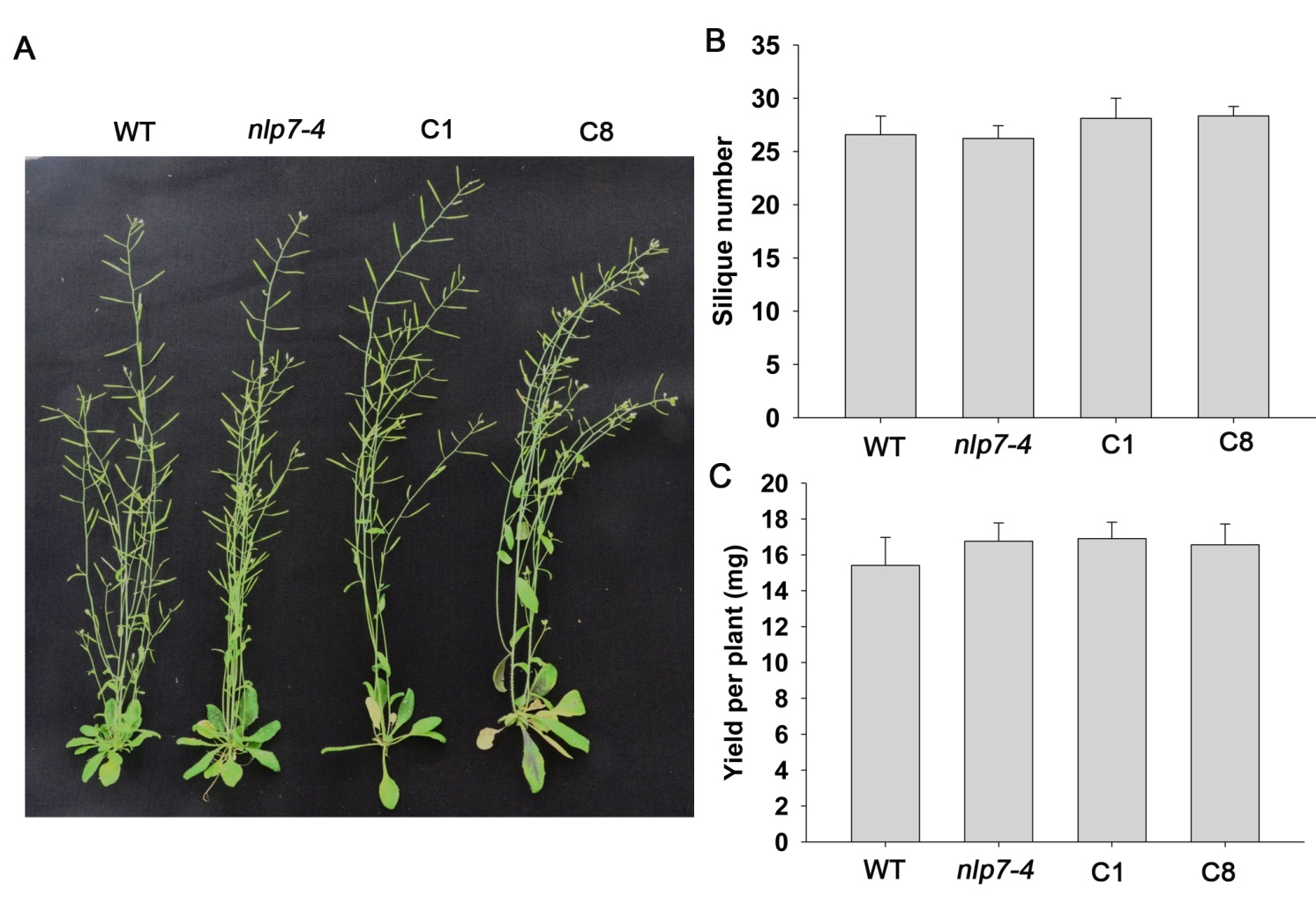


**Supplementary Figure 11** *ZmNLP6* and *ZmNLP8* did not increase yield per plant under high nitrate condition. (A) WT, *nlp7-4*, and *ZmNLP6* and *ZmNLP8* transgenic lines (C1: ZmNLP6/nlp7-4-1, C8: ZmNLP8/nlp7-4-8) grown in vermiculite watered with 5 mM KNO_3_ for 52 d. (B)The silique number and (C) yield per plant in transgenic lines Error bars represent SD of five biological replicates and each containing 9 plants, (u-test).

## 2. Supplementary Tables

**Table S1. The sequences of primers used in manuscript.**

| **Primer name** | **Sequence (**5'-3'**)** | **Application** |
| --- | --- | --- |
| Actin-F | GATTCCTGGGATTGCCGAT | qPCR |
| Actin-R | TCTGCTGCTGAAAAGTGCTGAG | qPCR |
| qZmNLP1-F | ccatgtacggcgaagaaaga | qPCR |
| qZmNLP1-R | ctcccgagcaatgccaaac | qPCR |
| qZmNLP2-F | ctgtggagagcagcagatgt | qPCR |
| qZmNLP2-R | aacactatcggtctctgcttca | qPCR |
| qZmNLP3-F | ttctcgaagcctgtcatccc | qPCR |
| qZmNLP3-R | gcactctgggtttagtcgga | qPCR |
| qZmNLP4-F | cagtgcgcagaattcaagga | qPCR |
| qZmNLP4-R | tggccacctcatctttcaaag | qPCR |
| qZmNLP5-F | tctgaaggaggccgcatg | qPCR |
| qZmNLP5-R | caccctgaacctgacgatgt | qPCR |
| qZmNLP6-F | tcatgccagagaagtccagc | qPCR |
| qZmNLP6-R | ggctctttattgaactcctctgg | qPCR/RT-PCR |
| qZmNLP7-F | ctgaaaaggctaatggtggaca | qPCR |
| qZmNLP7-R | tctgagccaaatgttgcctt | qPCR |
| qZmNLP8-F | GCGAAGAGGCTAAGGATGGA | qPCR/RT-PCR |
| qZmNLP8-R | TCCATGCATTCTTCCAAGTCTG | qPCR |
| qZmNLP9-F | ggttggaggttgctttccag | qPCR |
| qZmNLP9-R | aaacagtagctctcccgtcc | qPCR |
| ZmNLP6-F | CTGCAGGTACGATCACACTAGCACCGCCAGC | Cloning |
| ZmNLP6-R | GGTACCACCGGAGCTTCCACAAGAACTGCCA | Cloning |
| ZmNLP8-F | GGGTCGACGCAGCAGCAAGGTTTCATCCCAT | Cloning |
| ZmNLP8-R | GGGGTACCACCAGAGCTTCCACAAGAACTGC | Cloning |
| ZmNLP6GFP-F | ggtaccATATGGACCTCGACCCGGCCTAT | Localization assay |

**Continued table 1**

| **Primer name** | **Sequence** | **Application** |
| --- | --- | --- |
| ZmNLP6GFP-R | TCTTGTGGAAGCTCCGGTTGAgcggccgc | Localization assay |
| ZmNLP8GFP-F | ggtaccATATGGACTTCGACCCCT | Localization assay |
| ZmNLP8GFP-R | TTGTGGAAGCTCTGGTTGAgcggccgc | Localization assay |
| ZmNLP6Y1H-F | GATTATGCCTCTCCCGAATTCGTACGATCACACTAGCACCGCCAGC | Y1H Assay |
| ZmNLP6Y1H-R | CTTCTCGAGTCGGCCGAATTCACCGGAGCTTCCACAAGAACTGCCA | Y1H Assay |
| ZmNLP8Y1H-F | GATTATGCCTCTCCCGAATTCGCAGCAGCAAGGTTTCATCCCAT | Y1H Assay |
| ZmNLP8Y1H-R | CTTCTCGAGTCGGCCGAATTCACCAGAGCTTCCACAAGAACTGC | Y1H Assay |
| ZmNRT1.2NRE-F | AATTCCGCCCCCTCTATTTATCCAGGAGCTGCG | Y1H Assay |
| ZmNRT1.2NER-R | TCGACGCAGCTCCTGGATAAATAGAGGGGGCGG | Y1H Assay |
| ZmNiR2NRE-1-F | AATTCGGCCTCTTCCACCGCCGCAAGCACCAGG | Y1H Assay |
| ZmNiR2NRE-1-R | TCGACTGGCTCTTGGGGAGTTCAAGGGGGCAG | Y1H Assay |
| TUB2-F | ATCACGAACAGTGCCTTTGAACC | RT-PCR |
| TUB2-R | CAGCACCGACCTCTTCATAATCC | RT-PCR |
| 1300RT-R | CGTCATCGTCCTTGTAATCGA | RT-PCR |

**Table S2. The NRE motifs found in the nitrate related genes.**

|  | Gene | Gene ID | NRE Sequence |
| --- | --- | --- | --- |
| 1 | *ZmNiR2* | GRMZM2G079381 | NRE*^ZmNiR2-1^*: TGCCCCCTTgaactccccAAGAGCCA |
|  |  |  | NRE*^ZmNiR2-2^*: GGCCTCTTccaccgccgcAAGCACCAG |
| 2 | *ZmNIA3* | GRMZM2G428027 | NRE*^ZmNIA3^*: TGACCCTTccagcgcccAACGGCCTAT |
| 3 | *ZmNIA4* | GRMZM5G878558 | NRE*^ZmNIA4^*: TGGCCCTTggaggcggcgggAGGCGACC |
| 4 | *ZmNRT1.1A* | GRMZM2G086496 | NRE*^ZmNRT1.1A^*: CGACTTTTaaatgataaAAATATCC |
| 5 | *ZmNRT1.2* | GRMZM2G137421 | NRE*^ZmNRT1.2^*: CGCCCCCTctatttatccAGGAGCTGC |
| 6 | *ZmNRT2.1* | GRMZM2G010280 | NRE*^ZmNRT2.1^*: TGATCCTTggctgatcccACGGGAT |
| 7 | *ZmNRT2.5* | GRMZM2G455124 | NRE*^ZmNRT2.5-1^*: TTGCCCTTTgagaacccAGAGGTCGCG |
|  |  |  | NRE*^ZmNRT2.5-2^*: TGGCTCTTctccttcgcgAGGCCGC |

The upper case indicates the conserved nucleotides in NRE motif.
